# Studies on effects of static electric field exposure on liver in mice

**DOI:** 10.1038/s41598-018-33447-2

**Published:** 2018-10-19

**Authors:** Qinhao Lin, Li Dong, Yaqian Xu, Guoqing Di

**Affiliations:** Institute of Environmental Process, College of Environmental and Resource Sciences, Zhejiang University, Hangzhou, 310058 PR China

## Abstract

With the development of ultra-high-voltage direct-current transmission, the intensity of static electric field (SEF) under transmission lines increased, which has aroused public attention on its potential health effects. In order to examine effects of SEF exposure on liver, institute of cancer research mice were exposed to SEF with intensities of 27.5 kV/m, 34.7 kV/m and 56.3 kV/m, respectively. In each intensity of SEF exposure, a corresponding sham exposure group was used. Several indices relating to liver function (aspartate aminotransferase (AST) and alanine aminotransferase (ALT)) and oxidative stress (superoxide dismutase (SOD), glutathione peroxidase (GSH-Px) and malondialdehyde (MDA)) were tested after exposure of 7, 14, 21 and 35 days. Results showed that exposure to SEF with intensities of 27.5 kV/m and 34.7 kV/m for 35 days did not significantly influence any detected indices above. Under SEF exposure with intensity of 56.3 kV/m, the SOD activity in liver was significantly increased after exposure of 7 and 14 days. However, no significant increase was found in MDA content as well as the activities of AST and ALT between exposure group and sham exposure group during SEF exposure of 56.3 kV/m. It suggested that from three SEF intensities, only exposure to SEF with intensity of 56.3 kV/m (7 and 14 days) caused a temporary oxidative stress response in liver expressed by the increase in activity of SOD, but it did not produce oxidative damage. This biological effect may be related to the increase of mitochondrial membrane potential of hepatocytes caused by SEF exposure. When the membrane potential exceeds a threshold, Q cycle in mitochondria will be affected, which will result in an increase of superoxide anion concentration and ultimately an oxidative stress.

## Introduction

The energy distribution and demand in China are extremely unbalanced. Energy such as coal and petroleum are mainly located in the northwest while the demand is mainly concentrated in the southeast. In order to solve this problem, energy can be directly transported to the demand place by waterway or landway. A more effective way is converting energy into electricity *in situ*, which can be transported to the demand place over a long distance via ultra-high voltage transmission technology. Therefore, ultra-high-voltage direct-current (UHVDC) transmission technology with advantages of large transmission capacity and low energy loss has been rapidly developed recently in China^1^. At the same time, in order to promote the optimal allocation of global energy, the construction of the global energy internet has gradually become a consensus in the world. Hence, UHVDC transmission will be widely used in transnational and intercontinental power grid interconnection. UHVDC transmission can play an important role in promoting the global economic development and bringing convenience to our daily life. However, the potential health effects of static electric and magnetic field (0 Hz) from UHVDC transmission lines have aroused public concerns.

Research showed that the level of static magnetic field from UHVDC transmission lines was about 20 μT^2^ in the vicinity of ground, which was far less than the exposure limit of 400 mT in the *Guidelines on Limits of Exposure to Static Magnetic Fields* established by International Commission on Non-Ionizing Radiation Protection (ICNIRP). Therefore, the biological effects of static magnetic field from UHVDC transmission lines are negligible. As for static electric field (SEF), the maximum combined field intensity from ± 800 kV (the highest voltage level at present) UHVDC transmission line is about 20 kV/m~35 kV/m in the vicinity of ground^3–5^. Different from static magnetic field, there is no standard or guideline specified by international organizations to limiting the exposure of SEF. Although some countries such as China, American and Russia have formulated corresponding standards according to the developing situation of UHVDC transmission projects^6,7^, the exposure limits for SEF put forward by these countries vary largely from each other. And these standards are mainly based on the independent body feeling tests and electric shock tests, lacking support evidences from animal experiments of long-term exposure and epidemiological investigations. Therefore, in order to examine whether long-term SEF exposure will induce health risks, relevant animal experiments are greatly needed.

A number of researches^8–11^ have reported that power frequency electromagnetic field (50 Hz or 60 Hz) could induce oxidative stress and lead to oxidative damage by prolonging the lifespan of free radicals or increasing the intracellular concentration of free radicals. For example, Cui *et al*.^12^ found that the exposure of power frequency electromagnetic field could cause a serious oxidative stress in hippocampus and striatum, and could further impair corresponding ability about spatial learning and habit learning. A research from Yokus *et al*.^13^ showed that the exposure of power frequency electromagnetic field could induce oxidative DNA damage and lipid peroxidation expressed by the increase in the level of 8-hydroxy-2-deoxyguanosine and thiobarbituric acid reactive substances. Lai and Sing^14^ also reported that exposure to 60 Hz, 0.01 mT power frequency electromagnetic field could cause an oxidative damage in brain of rats as well as an increase in DNA single- and double-strand breaks. Since the frequency of static electric field is 0 Hz, its interaction mechanisms and biological effects are probably different from the 50 Hz or 60 Hz power frequency electromagnetic field. Therefore, it is worthy to examine and clarify whether SEF can also affect the lifespan or concentration of free radicals and induce an oxidative damage.

Liver is sensitive to oxidative stress due to having abundant mitochondria. Mitochondria are the main site producing reactive oxygen species (ROS) and are also the primary target attacked by ROS. A higher level of ROS can damage proteins, lipids and DNA, which may ultimately lead to cell death^15^. Moreover, as an important component of body’s antioxidant system, liver contains a variety of antioxidant enzymes, such as superoxide dismutase (SOD) and glutathione peroxidase (GSH-Px), which can regulate the overall level of ROS to maintain physiological homeostasis^16^. Therefore, an oxidative damage of liver induce by SEF can be examined partially through detecting oxidative stress indices (such as SOD and GSH-Px).

In this study, a self-made high-voltage device producing SEF was used to study the effects of long-term (lasting 35 days) SEF exposure on liver, and several typical oxidative stress indices and liver function indices in mice were detected under SEF exposure of 27.5 kV/m, 34.7 kV/m and 56.3 kV/m, respectively.

## Materials and Methods

### Experimental animals

Healthy male Institute of Cancer Research (ICR) mice (4 weeks old and weighted 19.6 ± 1.6 g) obtained from the Experimental Animal Center of Zhejiang Province (Hangzhou, China) were used for experiments. Three experiments were carried out independently. In each experiment, the intensities of SEF were different and the exposure duration was all 35 days, during which 4-week-old mice went through their preadolescent phase (about postnatal day 30 to day 45) and adolescent phase (about postnatal day 45 to day 60)^17^. In each experiment, 80 mice were used and were randomly divided into two groups: SEF exposure group (*n* = 40) and sham exposure group (*n* = 40). After 3 days lasting adaptation in laboratory, mice in SEF exposure group began to receive a 24 hours lasting exposure of SEF. The mice were kept under controlled temperature (22 ± 2 °C), humidity (50~60%) and a 12 h/12 h light/dark cycle (light on from 08:00 to 20:00), and they had free access to food and water. All animal experimental procedures were performed in accordance with Quality Management Approach to Laboratory Animals and were approved by the Animal Care and Use Committee of Zhejiang University (Permission No.12923). Every possible effort was made to minimize the number of animals used and their suffering.

### Exposure to static electric field

**Figure 1:**
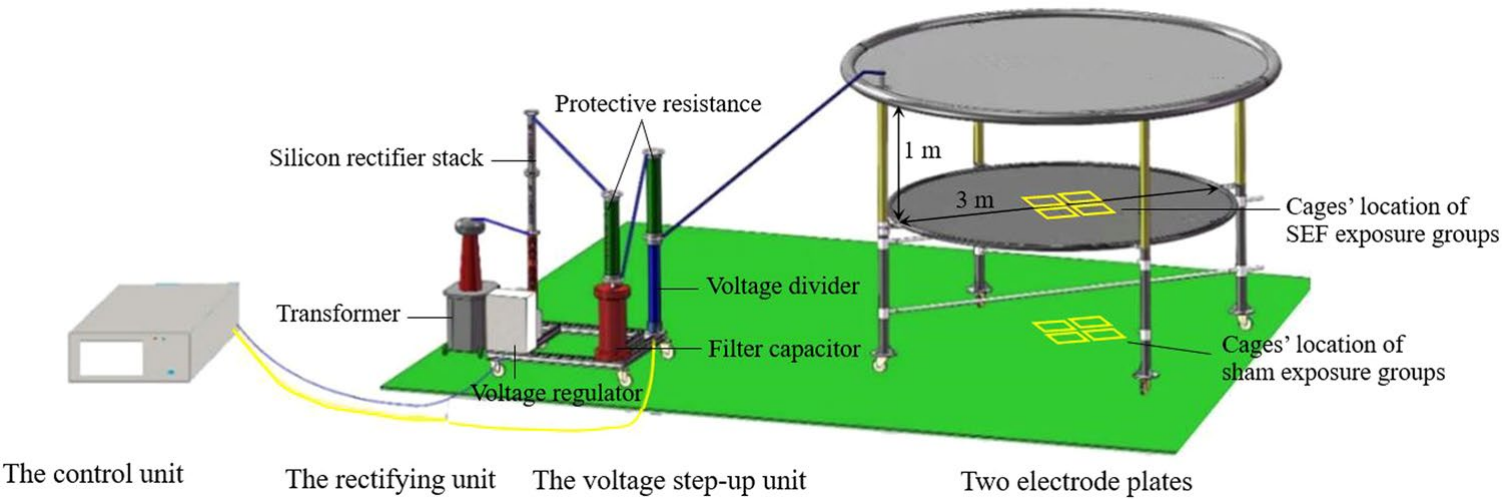
Schematic diagram of the high-voltage device used for SEF exposure.

The exposure to SEF was performed using the self-made high-voltage device described by Wu *et al*.^18^. Specifically, the device was mainly composed of four parts, two electrode plates, a voltage step-up unit, a rectifying unit and a control unit, as shown in Fig. 1^19^. The two electrode plates were arranged up and down in parallel with a distance of 1 m. A uniform SEF was generated between two electrode plates and the maximal output voltage could be 100 kV.

All experimental mice were housed in specially made polycarbonate cages (35 cm × 25 cm × 46 cm, length × width × height) with an open top. The SEF exposure group was placed at the center of the bottom electrode plate for SEF exposure (Fig. 1), while the sham exposure group was placed on the floor, right under the exposure group (since the bottom electrode was well grounded, the theoretical intensity of SEF in the space between the bottom electrode plate and the floor was 0 kV/m). The exposure conditions of the mice in sham exposure group were the same as those in SEF exposure group except that they did not receive SEF exposure. In addition, considering the interference of the power frequency electromagnetic field generated by the device itself, the background levels of power frequency electric and magnetic fields were also measured. Results showed that the average levels of power frequency electric and magnetic fields were about 0.06 kV/m and 0.04 μT at both the positions of the exposure and sham exposure groups, which were far below the limits of corresponding standard and can be ignored.

Three independent SEF exposure experiments were carried out successively, and the theoretical values of SEF intensities were 40 kV/m, 50 kV/m and 80 kV/m, respectively. That is, the voltage applied between the two electrodes were 40 kV, 50 kV and 80 kV, respectively. Actually, the experimental animals were housed in cages whose sizes, materials and shapes would affect the distribution of electric field inside. Therefore, in order to measure the actual exposure intensity affecting mice, a SEF intensity detection system (HDEM-1, Beijing Safety Test Technology Co., Ltd., China) with an isotropic SEF probe (diameter, 8 cm) was used to measure the SEF intensity inside the cages of exposure and sham exposure group (at the center of cage, about 8 cm above the bottom). Measuring results indicated that when the theoretical SEF intensity was 40 kV/m, 50 kV/m and 80 kV/m, the actual SEF intensity (mean ± SD) inside cages was 27.5 ± 1.0 kV/m (mean ± SD), 34.7 ± 1.1 kV/m and 56.3 ± 1.4 kV/m, respectively. The SEF intensity of 27.5 kV/m was close to the exposure limits of SEF specified in existing local or industry standards^6,7,19^; 34.7 kV/m was almost the maximum intensity of SEF in the vicinity of ground generated by actual UHVDC transmission lines^3–5^; 56.3 kV/m was a relatively high intensity and was about 2 times higher than the minimum intensity used in this study. Furthermore, results also showed that with the increase of voltage applied between two electrodes, the intensity of SEF inside the cages of sham exposure group had almost no change and the intensity was 0.28 ± 0.01 kV/m, which was close to the background level of SEF (0.1~0.3 kV/m)^20^.

### Determination of hepatic oxidative stress indices

After 7, 14, 21 and 35 days exposure of SEF, mice from both groups (10 mice per group) were used for the determination of hepatic oxidative stress indices. The mice were anesthetized by ether and sacrificed by decapitation. Then the liver tissues was dissected quickly on an ice plate. About 0.2 g liver samples were homogenized in cold saline at a ratio of 1:9 (mass/volume) by an automatic sample grinder (Tissuelyser-48, China). After centrifugations (3500 r/min), the supernatants of the liver homogenate were obtained and analysis kits (Nanjing Jiancheng Bioengineering Inst., Nanjing, China) were used for determination^21^. The activities of SOD and GSH-Px as well as the content of malondialdehyde (MDA) in supernatants were detected by spectrophotometric method.

### Determination of liver function indices

After 7, 14, 21 and 35 days exposure of SEF, mice from both exposure group and sham exposure group (10 mice per group) were used for the determination of liver function indices. Approximately 1 mL of blood was sampled from mice by eyeball extirpating, which was more convenient and easier than cardiac puncture to collect the blood of mice. Then, the blood sample was stood for 1 hour at room temperature. After the serum was precipitated, the blood sample was centrifuged for 20 minutes (3000 r/min) by a high-speed centrifuge. The supernatant was used to determine liver function indices including alanine aminotransferase (ALT) and aspartate aminotransferase (AST) by a blood biochemical analyzer (Cobas c311, Switzerland).

### Statistical analysis

All results were presented as mean values ± SD for each group. All data were obeyed the normal distribution and their variance were homogeneous, so the difference between exposure group and sham exposure group was analyzed by independent-samples *t* test. A value of *p* < 0.05 was considered to be statistically significant and was presented by *. Furthermore, power analysis was also conducted using PASS 11.0.

## Results

### Activity of SOD in liver

**Figure 2:**
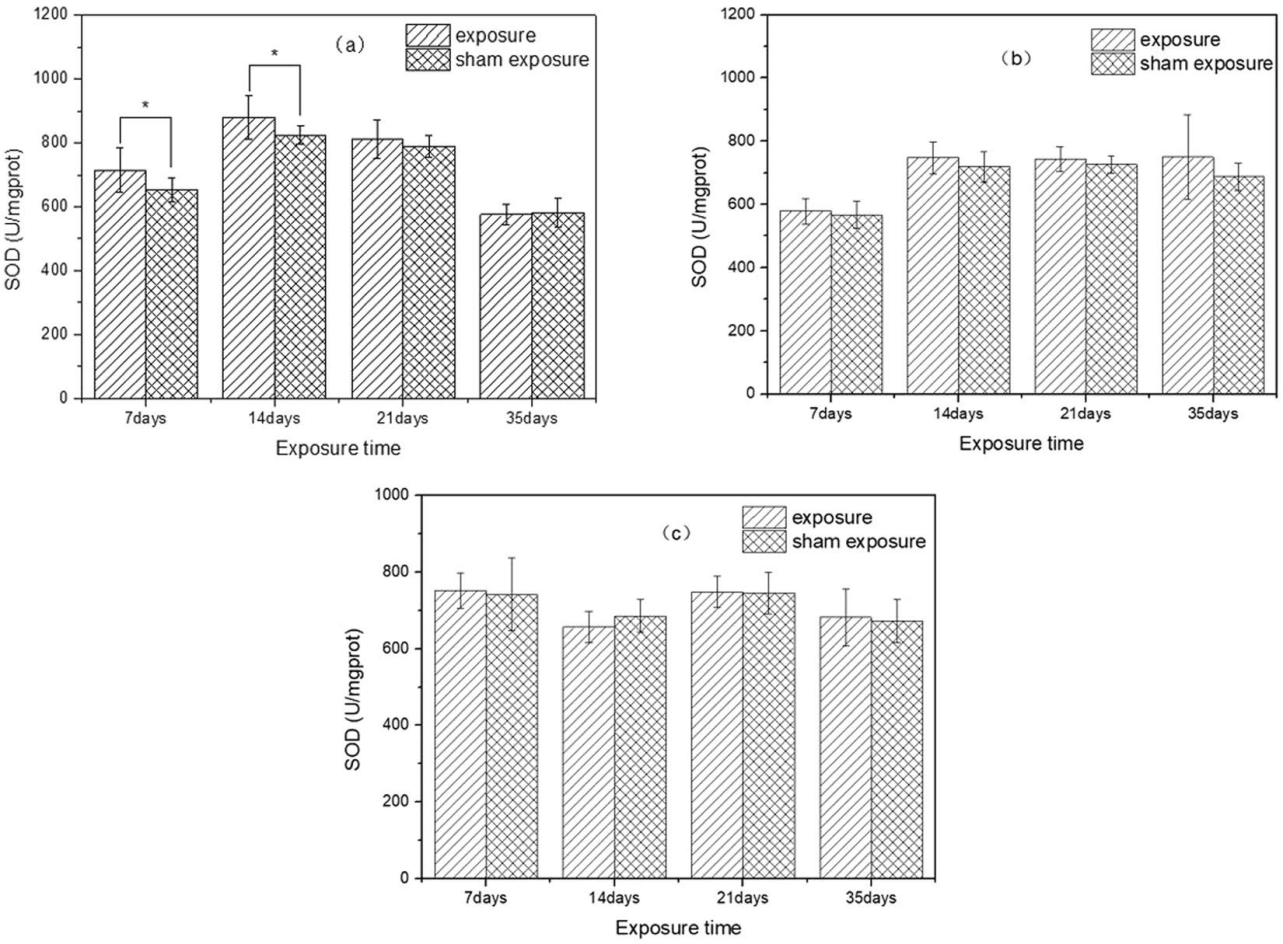
Effects of SEF on SOD activity in the liver of mice. (**a**) 56.3 kV/m, (**b**) 34.7 kV/m, (**c**) 27.5 kV/m.

The results of SOD activity in liver after exposure to SEF with the intensities of 56.3 kV/m, 34.7 kV/m, 27.5 kV/m and corresponding sham exposure were shown in Fig. 2. Under exposure to SEF with intensity of 56.3 kV/m, the SOD activity in exposure group was significantly higher than that in sham exposure group on the 7^th^ and 14^th^ days of exposure (7 days, *p* = 0.02; 14 days, *p* = 0.03). However, there was no significant difference between exposure group and sham exposure group on the 21^th^ and 35^th^ days of exposure (21 days, *p* = 0.30; 35 days, *p* = 0.73). Moreover, under exposure to SEF with intensities of 34.7 kV/m and 27.5 kV/m, there were no significant differences in SOD activity between exposure groups and sham exposure groups (SEF exposure of 34.7 kV/m (7 days: *p* = 0.56; 14 days: *p* = 0.20; 21 days: *p* = 0.29; 35 days: *p* = 0.18); SEF exposure of 27.5 kV/m (7 days: *p* = 0.77; 14 days: *p* = 0.14; 21 days: *p* = 0.91; 35 days: *p* = 0.76)). Post-hoc power analysis showed that the statistical power of SOD results were all lower than 0.8.

### Activity of GSH-Px in liver

**Figure 3:**
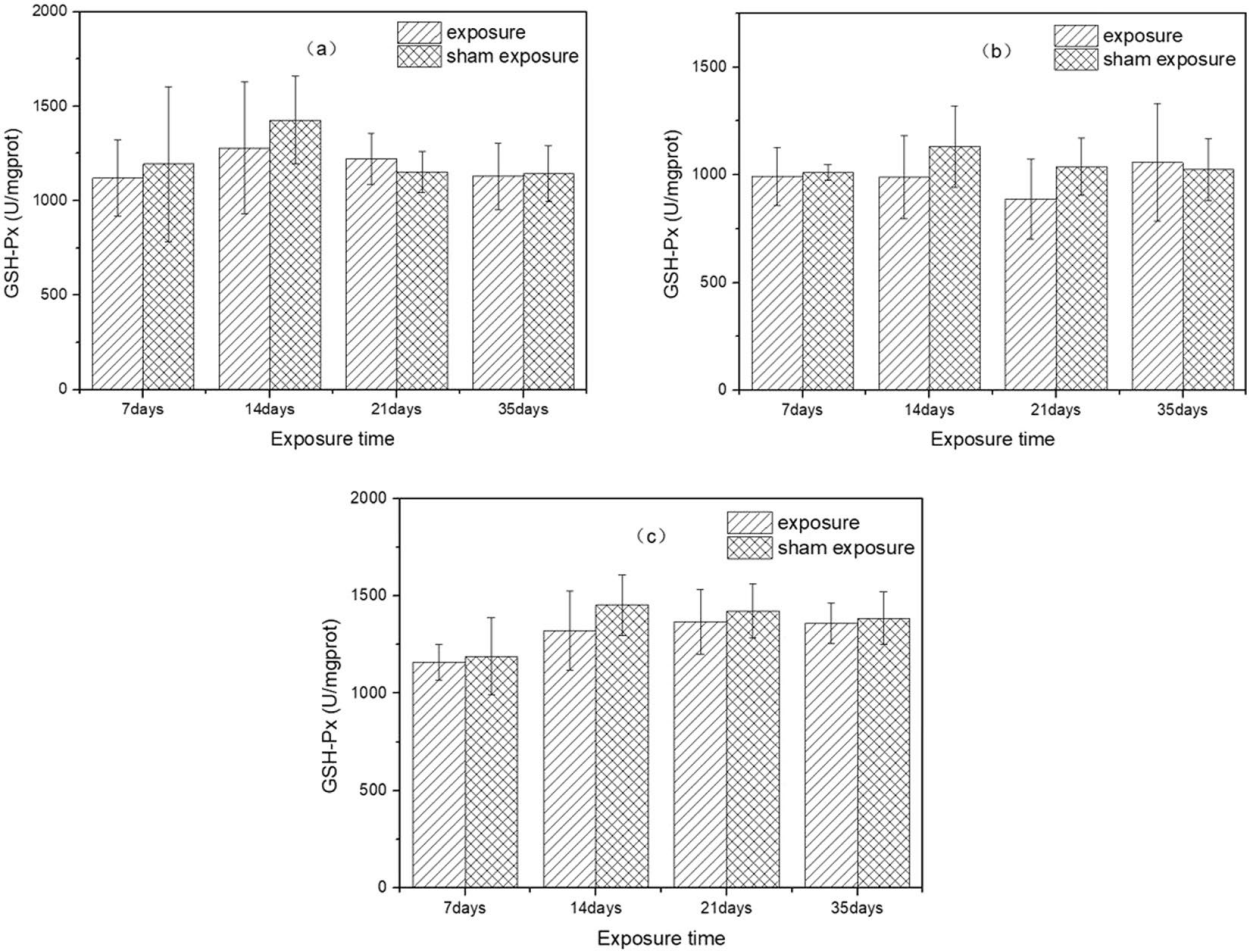
Effects of SEF on GSH-Px activity in the liver of mice. (**a**) 56.3 kV/m, (**b**) 34.7 kV/m, (**c**) 27.5 kV/m.

The results of exposure to SEF with the intensities of 56.3 kV/m, 34.7 kV/m, 27.5 kV/m and corresponding sham exposure on the activity of GSH-Px in liver were presented in Fig. 3. There were no significant differences in GSH-Px activity between exposure groups and sham exposure groups on any analyzed day of SEF exposure with three different intensities (SEF exposure of 56.3 kV/m (7 days: *p* = 0.62; 14 days: *p* = 0.28; 21 days: *p* = 0.22; 35 days: *p* = 0.85); SEF exposure of 34.7 kV/m (7 days: *p* = 0.75; 14 days: *p* = 0.11; 21 days: *p* = 0.07; 35 days: *p* = 0.75); SEF exposure of 27.5 kV/m (7 days: *p* = 0.67; 14 days: *p* = 0.12; 21 days: *p* = 0.44; 35 days: *p* = 0.67)). Post-hoc power analysis showed that the statistical power of GSH-Px results were all lower than 0.8.

### Content of MDA in liver

**Figure 4:**
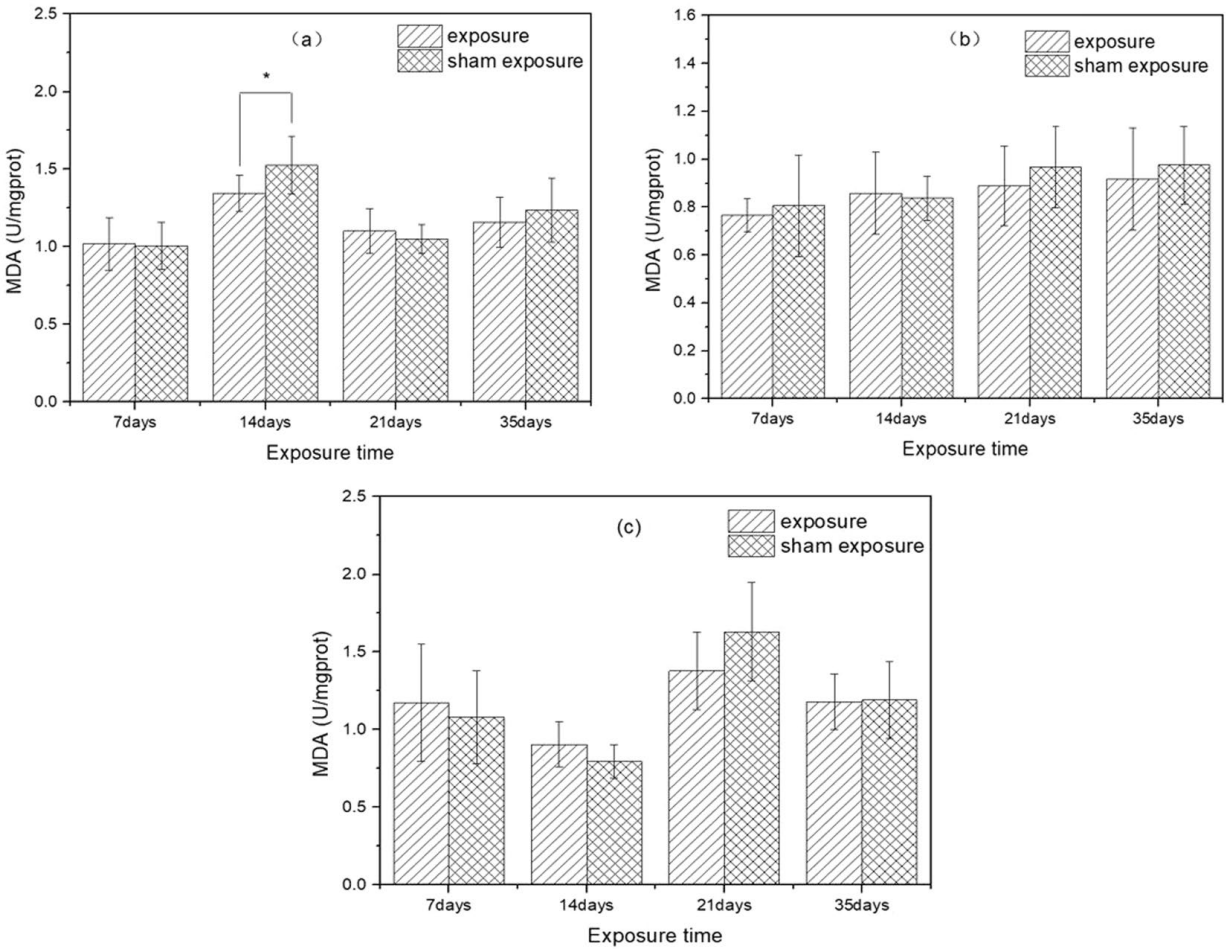
Effects of SEF on MDA content in the liver of mice. (**a**) 56.3 kV/m, (**b**) 34.7 kV/m, (**c**) 27.5 kV/m.

The results of MDA content in the liver of mice in exposure groups and sham exposure groups were presented in Fig. 4. After exposure to SEF with intensity of 56.3 kV/m for 14 days, the MDA content in exposure group was significantly lower than that in sham exposure group (*p* = 0.02), while there was no significant difference between the exposure group and corresponding sham exposure group on 7^th^, 21^st^ and 35^th^ day of exposure (7 days: *p* = 0.85; 21 days: *p* = 0.36; 35 days: *p* = 0.36). Besides, there were no significant differences in MDA content between mice from groups exposed to SEF with intensities of 34.7 kV/m or 27.5 kV/m and mice from corresponding sham exposure groups (SEF exposure of 34.7 kV/m (7 days: *p* = 0.57; 14 days: *p* = 0.75; 21 days: *p* = 0.31; 35 days: *p* = 0.51); SEF exposure of 27.5 kV/m (7 days: *p* = 0.54; 14 days: *p* = 0.07; 21 days: *p* = 0.08; 35 days: *p* = 0.90)). Post-hoc power analysis displayed that the statistical power of MDA results were all lower than 0.8.

### Activity of ALT in serum

**Figure 5:**
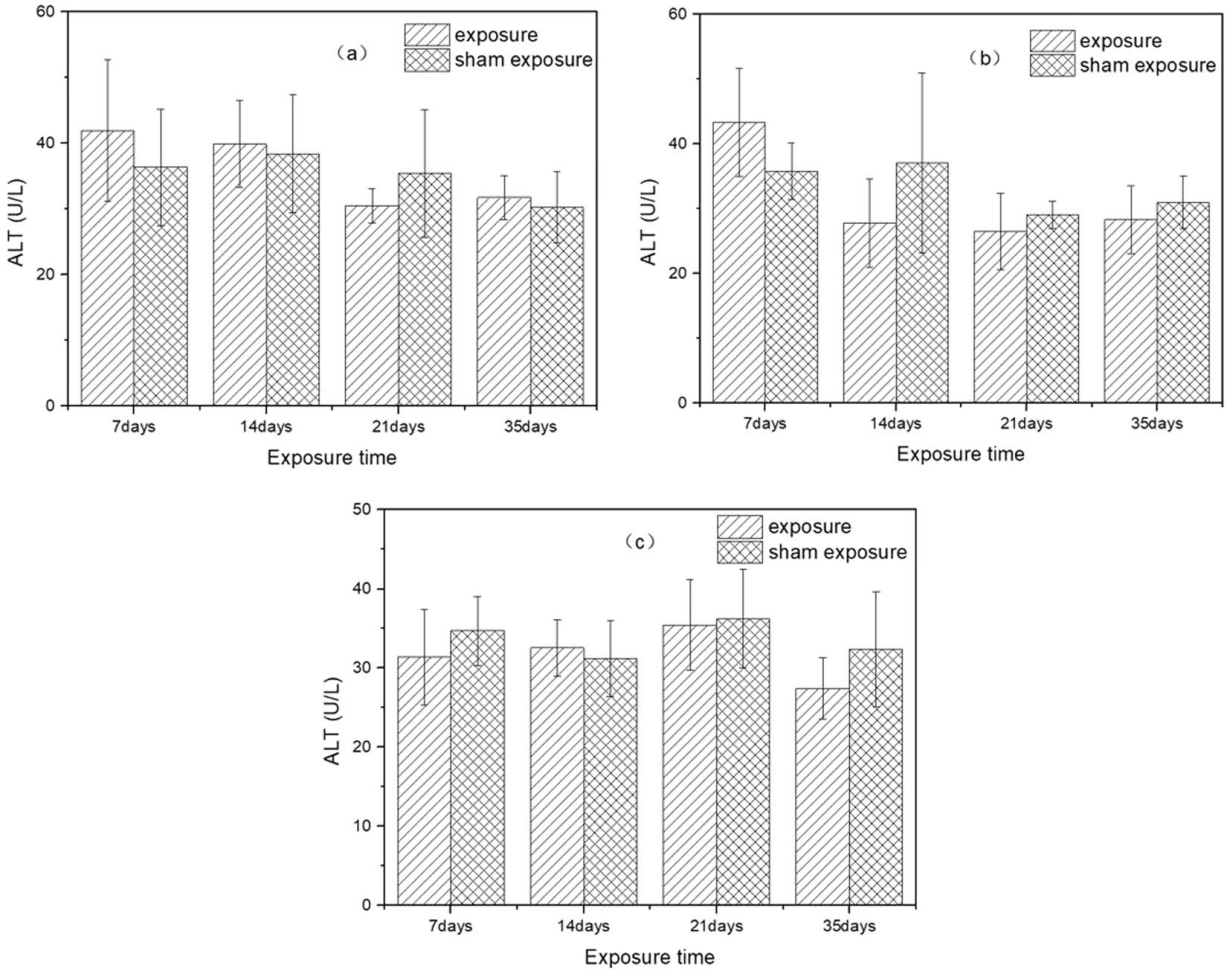
Effects of SEF on ALT activity in the serum of mice. (**a**) 56.3 kV/m, (**b**) 34.7 kV/m, (**c**) 27.5 kV/m.

The results of serum ALT activity after exposure to SEF with the intensities of 56.3 kV/m, 34.7 kV/m, 27.5 kV/m and corresponding sham exposure were shown in Fig. 5. There were no significant differences in the serum activity of ALT between mice from groups exposed to SEF with three different intensities and corresponding sham exposure groups (SEF exposure of 56.3 kV/m (7 days: *p* = 0.34; 14 days: *p* = 0.45; 21 days: *p* = 0.26; 35 days: *p* = 0.59); SEF exposure of 34.7 kV/m (7 days: *p* = 0.08; 14 days: *p* = 0.17; 21 days: *p* = 0.33; 35 days: *p* = 0.26); SEF exposure of 27.5 kV/m (7 days: *p* = 0.33; 14 days: *p* = 0.61; 21 days: *p* = 0.83; 35 days: *p* = 0.17)). Post-hoc power analysis showed that the statistical power of ALT results were all lower than 0.8.

### Activity of AST in serum

**Figure 6:**
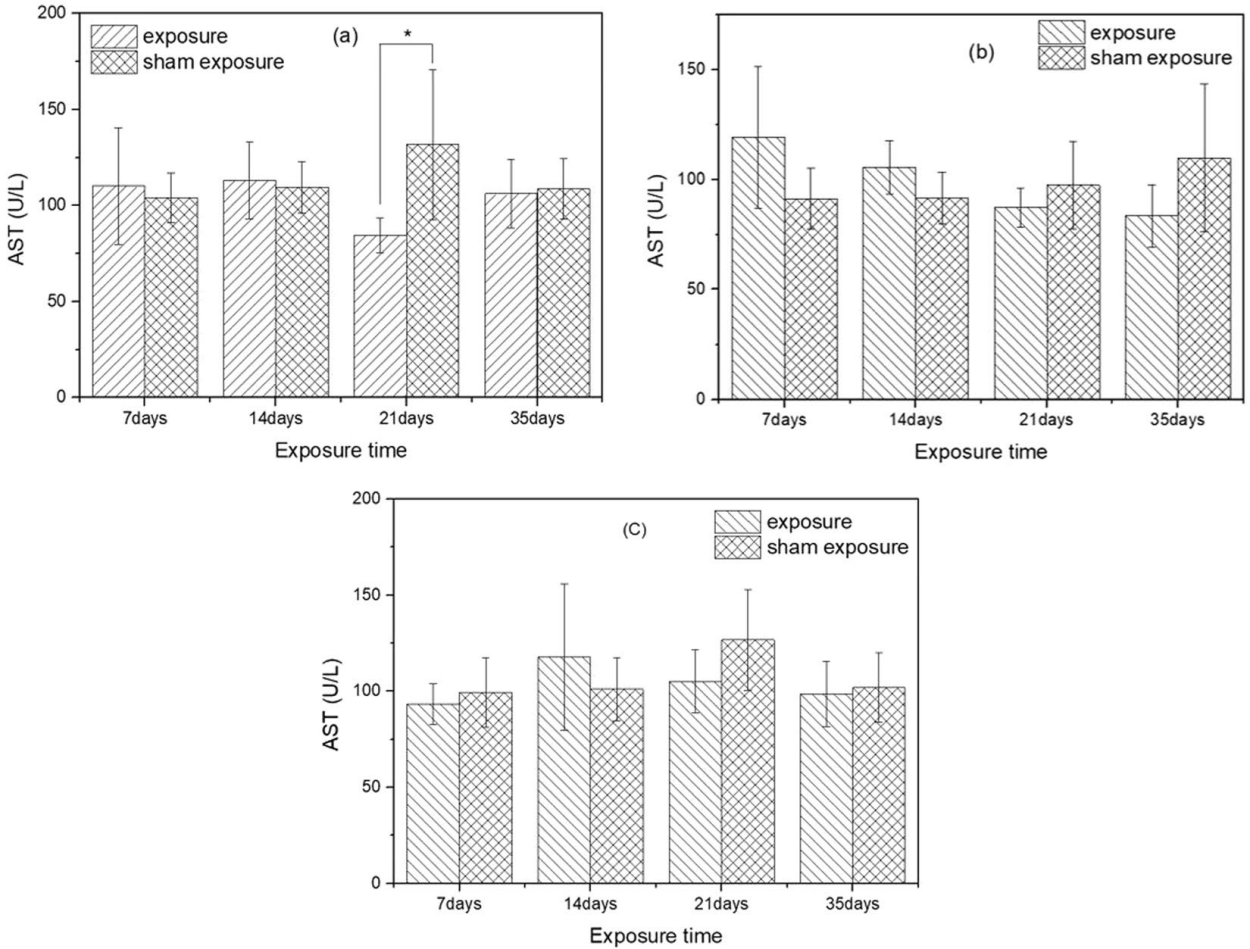
Effects of SEF on AST activity in the serum of mice. (**a**) 56.3 kV/m, (**b**) 34.7 kV/m, (**c**) 27.5 kV/m.

The results of serum AST activity after exposure to SEF with the intensities of 56.3 kV/m, 34.7 kV/m, 27.5 kV/m and corresponding sham exposure were presented in Fig. 6. After exposure to SEF with intensity of 56.3 kV/m for 21 days, the activity of AST in exposure group was significantly lower than that in sham exposure group (*p* = 0.02). However, there were no significant differences in serum AST activity between the exposure groups and corresponding sham exposure groups after 7 days, 14 days and 35 days of exposure (7 days: *p* = 0.66; 14 days: *p* = 0.73; 35 days: *p* = 0.80). Moreover, there were no significant differences in the activity of ALT between mice from groups exposed to SEF with intensities of 34.7 kV/m, 27.5 kV/m and corresponding sham exposure groups (SEF exposure of 34.7 kV/m (7 days: *p* = 0.08; 14 days: *p* = 0.08; 21 days: *p* = 0.27; 35 days: *p* = 0.55); SEF exposure of 27.5 kV/m (7 days: *p* = 0.50; 14 days: *p* = 0.35; 21 days: *p* = 0.12; 35 days: *p* = 0.76)). Post-hoc power analysis showed that only after exposure to SEF with intensity of 56.3 kV/m for 21 days, the AST result showed a large power (>0.8). And the statistical power of other results were all lower than 0.8.

## Discussions

The release of enzymes in liver is an important indicator of the hepatocytes damage, in which ALT and AST are the two most sensitive indices^22,23^. ALT and AST are both intracellular enzymes and normally their activities are low in serum. When hepatocytes are damaged, the permeability of cell membrane will increase. Accordingly, the ALT and AST inside the cell will pour into the blood, causing a substantial increase of their activities in serum^24^. Thus, the activities of ALT and AST in serum can directly reflect the damage of hepatocytes. Güler *et al*.^25^ studied the effect of exposure to SEF with intensities of 0.3~1.8 kV/m on serum enzymes activities of guinea pig and found that 3 days exposure had no significant effect in the activities of ALT, alkaline phosphatase, lactate dehydrogenase and other liver function indices. Similarly to the result of Güler *et al*., in this study, after 7~35 days of exposure to SEF with the intensities of 27.5 kV/m, 34.7 kV/m and 56.3 kV/m, no significant increase was observed in activities of AST and ALT in the serum of mice. It suggested that when the intensity of SEF was at 56.3 kV/m or below, exposure lasting 35 days would not cause damage to hepatocytes.

Oxidative stress refers to the imbalance between production and elimination of free radicals (such as superoxide radicals, hydrogen peroxide, hydroxyl radicals, *etc*.), which will result in the accumulation of ROS *in vivo* or in cells and potentially lead to oxidative damage^26^. Superoxide anion (O_2_^−^∙), arising either through metabolic processes or following oxygen “activation” by physical irradiation, is considered as the primary ROS^27^. Superoxide anion not only has cytotoxicity in itself but also can interact with other molecules to generate secondary ROS (such as HO_2_∙, H_2_O_2_, OH∙, *etc*.) causing a further oxidative damage to organism. Generally, superoxide anion *in vivo* can be converted into O_2_ and H_2_O_2_ by SOD, and the latter can be further metabolized into H_2_O by GSH-Px and other scavenging enzymes. Thus, SOD and GSH-Px play a vital role in body’s antioxidant defense, and their activities are considered as a reliable biomarker of ROS contents and intensity of oxidative stress^28^. ROS accumulated *in vivo* will attack the polyunsaturated fatty acid of biomembrane and produce lipid peroxides such as MDA^29^. Hence, the content of MDA can reflect the level of oxidative damage in cells. Many studies showed that oxidative stress is a crucial cause for the damage of liver function *in vivo* or in cells induced by the exposure of electromagnetic fields^24,29^. Li *et al*.^24^ exposed Wistar rats to 50-Hz electromagnetic field for 10 weeks and found that their liver function were impaired, which was related to the oxidative stress induced by electromagnetic field. Emre *et al*.^30^ found that the exposure to power frequency electromagnetic field could increase levels of SOD and MDA in rats and lead to an oxidative damage of liver tissues. In this study, the activity of SOD in exposure group was increased significantly after exposure to SEF with intensity of 56.3 kV/m lasting 7 and 14 days. In general, a sharp increase of O_2_^−^∙ contents caused by external stimuli will result in oxidative stress in organism. In the meanwhile, organism can produce compensatory respond by activating or regulating corresponding biological pathways, that is, organism increase the synthesis of SOD to reestablish the homeostasis of O_2_^−^∙ *in vivo*^31–33^. When a high concentration of O_2_^−^∙ induced by a high-does stimuli keeps a long time in organism, the consumption rate of SOD will far exceed its synthesis rate, which macroscopically manifests as a reduction of SOD activity. Under low-does stimuli, the synthesis rate of SOD may be greater than its consumption rate through body’s overcompensation, which can lead to the increase of SOD activity during a short time. In this study, the increase of SOD activity, as a specific enzyme of O_2_^−^∙, in the liver of mice suggested that exposure to SEF with intensity of 56.3 kV/m could cause an increase of O_2_^−^∙ concentration and produce oxidative stress response. Nevertheless, during exposure to SEF with intensity of 56.3 kV/m, there was no significant increase in MDA content, as an indicator of oxidative damage. It suggested that although the exposure to SEF induced a temporary oxidative stress response in liver, it did not result in an oxidative damage, which was consistent with the test results of liver function indices (ALT and AST) in this study. A possible reason for this result is fact that the liver antioxidative capacity of mice was enhanced through the overcompensation of body, which prevented the liver from an obvious oxidative damage. Under further exposure to SEF with intensity of 56.3 kV/m (on 21^st^ and 35^th^ day), there was no significant difference in the activity of SOD in liver between exposure group and sham exposure group, which indicated that the synthesis of SOD was reduced by self-regulation and a new homeostasis of O_2_^−^∙ was reestablished.

Güler *et al*.^34^ hypothesized that SEF may promote the conversion of molecular O_2_ into O_2_^−^∙ and cause oxidative stress through the energy of electric field transferred in tissues. This hypothesis was consistent with the increase of SOD activity in mice after short-term exposure of SEF in this study. Research of Schapira^35^ shows that mitochondrial respiratory chain produces 95% of O_2_^−^∙ in oxidative metabolism of organism. Does the energy of electric field penetrating into body promote the conversion of O_2_^−^∙ by influencing this process? Destefanis *et al*.^36^ found that electromagnetic fields can increase the membrane potential of mitochondrial in several kinds of cancer cells and lead to an enhancement of mitochondrial activity. Wu *et al*.^18^ exposed male mice to SEF with intensity of 56 kV/m for 49 days and found a loss of cristae in some mitochondria of spermatogenic cells, which could directly change the mitochondrial membrane potential. Iorio *et al*.^37^ also reported that the exposure of electromagnetic field could increase the mitochondrial membrane potential in sperm. Obviously, the exposure of electromagnetic field could change the mitochondrial membrane potential of different cells. Liu^38^ showed that when the mitochondrial membrane potential exceeds a threshold, the cytochrome b_566_ can not be oxidized by b_562_. Accordingly, the semiquinone (QH∙) in Q cycle become long-lived in the Q_0_ center and more readily donate a single electron to oxygen to generate O_2_^−^∙, which will ultimately results in an increase of O_2_^−^∙ concentration in cells. Therefore, a possible mechanism of biological effects of SEF exposure could be related to the increase of O_2_^−^∙ concentration and could lead to development of oxidative stress by influencing the mitochondrial membrane potential of organism. Of course, more experiments should be designed and be carried out to verify this biological mechanism. In this study, under exposure to SEF with intensities of 27.5 kV/m and 34.7 kV/m, there were no significant differences in all hepatic oxidative stress indices between exposure groups and corresponding sham exposure groups. Probably, the cause of this result is that SEF with intensities of 27.5 kV/m and 34.7 kV/m were both not strong enough to cause membrane potential of mitochondria exceeding threshold and induce oxidative stress in mice. However, Cieslar *et al*.^39^ found that SEF with similar intensities (25 and 35 kV/m) could cause compensatory and multidirectional changes in the activities of various antioxidant enzymes (SOD, GSH-Px and catalase) in rats liver on the 14^th^ and 28^th^ day of exposures (8 hours/day). The difference of two results could be mainly attribute to the variance in experimental animals and daily exposure duration.

The indirect influence of surface charges, such as piloerection and cutaneous stimulation, is another possible explanation of SEF biological effects^20^. When organisms were exposed to SEF, not only polarized charges will come into being on the surface of skin but also charged ions in body fluids will migrate directionally to the surface of skin. With the increase of SEF intensity, surface charges including polarized charges and charged ions above will accumulate on the surface of skin. Ultimately, it will cause piloerection and cutaneous stimulation, and further lead to anxiety and other psychological effects. This phenomenon is evident in mice and other hirsute animals particularly^40^. The perception thresholds of different organisms for this stimulation caused by surface charges were different. Blondin *et al*.^41^ studied the perception threshold of humans staying under the high-voltage direct current transmission lines and found the average perception threshold was 45.1 kV/m. Creim *et al*.^42^ reported that during the exposure to SEF with intensity of 55 kV/m or greater, rats showed an obvious avoidance behavior, while such behavior did not occur during exposure to SEF with intensity of 42.5 kV/m or less, which indicated the perception threshold of SEF in rats was between 42.5 kV/m to 55 kV/m. Mice and rats are both hirsute animals and their perception thresholds of SEF could be similar approximately. Thus, the intensities of SEF of 27.5 kV/m and 34.7 kV/m could be both below the perception threshold of mice in this study, which was another possible reason why SEF with those intensities did not significantly influence the liver function indices and hepatic oxidative stress indices in mice.

Of course, it should be noted that the lack of power in results of this study probably led to that non-significant difference between the exposure group and sham exposure group was false negative result. It could be further confirmed through additional animal experiments with a larger sample size.

## Conclusions

1. From three analyzed intensities of SEF, only exposure to highest intensity of 56.3 kV/m SEF for a short time (7and 14 days) could induce a certain oxidative stress response in the liver of mice expressed by increase in activity of SOD, which was probably pertinent to the increase of mitochondrial membrane potential caused by the exposure of SEF in liver. When the membrane potential exceeds a certain threshold, it will affect the Q cycle in mitochondria and result in an increase of O_2_^−^∙ concentration, which will ultimately lead to a development of oxidative stress. Under exposure to SEF with lower intensities of 27.5 kV/m and 34.7 kV/m, the increment of liver mitochondrial membrane potential could be below the threshold, thus in those case SEF did not lead to a development of oxidative stress and liver tissue damage in mice.
2. Exposure to SEF with intensity of 56.3 kV/m, in spite of temporary oxidative stress response in the liver of mice appearing only in the initial phase of exposure (first 14 days), did not cause an obvious oxidative damage. This may be related to fact that the antioxidative capacity of liver was enhanced through body’s overcompensation under longer SEF stimulation.
3. The effects of SEF generated by actual UHVDC transmission lines on liver will be probably weaker than that of SEF with intensity of 56.3 kV/m analyzed in this study, as the intensities of such SEF in the vicinity of ground are smaller than that value.
4. More experiments with a larger sample size should be designed to verify the effect of SEF exposure on liver function, especially on mitochondrial membrane potential and its threshold, with special regard to longer exposure time exceeding 35 days.
